# How can interventions more directly address drivers of unprofessional behaviour between healthcare staff?

**DOI:** 10.1136/bmjoq-2024-002830

**Published:** 2024-07-08

**Authors:** Justin A Aunger, Ruth Abrams, Russell Mannion, Johanna I Westbrook, Aled Jones, Judy M Wright, Mark Pearson, Jill Maben

**Affiliations:** 1 Midlands Patient Safety Research Collaboration, Institute of Applied Health Research, University of Birmingham, Birmingham, UK; 2 School of Health Sciences, Faculty of Health and Medical Sciences, University of Surrey, Guildford, UK; 3 Health Services Management Centre, University of Birmingham, Birmingham, UK; 4 Australian Institute of Health Innovation, Macquarie University, Sydney, New South Wales, Australia; 5 School of Nursing and Midwifery, Faculty of Health, University of Plymouth, Plymouth, UK; 6 School of Medicine, Faculty of Medicine and Health, University of Leeds, Leeds, UK; 7 Wolfson Palliative Care Research Centre, Hull York Medical School, University of Hull, Hull, UK

**Keywords:** Professional Role, Safety culture, Patient safety, Quality improvement, Implementation science

## Abstract

Unprofessional behaviours (UBs) between healthcare staff are widespread and have negative impacts on patient safety, staff well-being and organisational efficiency. However, knowledge of how to address UBs is lacking. Our recent realist review analysed 148 sources including 42 reports of interventions drawing on different behaviour change strategies and found that interventions insufficiently explain their rationale for using particular strategies. We also explored the drivers of UBs and how these may interact. In our analysis, we elucidated both common mechanisms underlying both how drivers increase UB and how strategies address UB, enabling the mapping of strategies against drivers they address. For example, social norm-setting strategies work by fostering a more professional social norm, which can help tackle the driver 'reduced social cohesion'. Our novel programme theory, presented here, provides an increased understanding of what strategies might be effective to adddress specific drivers of UB. This can inform logic model design for those seeking to develop interventions addressing UB in healthcare settings.

## Introduction

Unprofessional behaviours (UBs) between staff can include, but are not limited to, microaggressions, incivility, bullying and harassment.^1^ These behaviours have negative impacts on staff well-being, patient safety, organisational reputation and organisational costs^2^ and are unfortunately prevalent in healthcare systems worldwide.^1 3 4^ We recently published two papers from our recent realist review. One reported a programme theory (PT) explaining five types of key driver of UBs in acute care settings and how these work^5^. The other reported a PT drawing on 42 reports of interventions using 13 types of behaviour change strategies to reduce UB.^6^ To improve the effectiveness of interventions to reduce UB, we found that it is essential to directly target drivers of UB with strategies that address them.^6^ However, which strategies best address particular drivers of UB have not yet been articulated.^7 8^ This report sets out which behaviour change strategies address specific drivers of UB based on common underlying mechanisms of action.

## Methods

Realist reviews seek to understand why an intervention may work (or not), for whom, in which contexts and why, through the generation of PTs using retroductive logic.^9^ These are generally depicted as context–mechanism–outcome (CMO) configurations.^10^ These mechanisms, in realist terms, can be defined as ‘changes in recipient reasoning that occur in response to resources introduced by an intervention’.^11^

In line with RAMESES guidelines,^9 10^ our first step was to build initial PTs by analysing 38 reports from organisations such as National Health Service (NHS) England, the King’s Fund and NHS Employers using NVivo V.12 for data organisation.^12 13^ We then tested and refined these theories against 110 additional studies (to December 2022) identified with systematic searches of Embase, CINAHL and MEDLINE databases, and grey literature repositories. Article selection involved screening records for inclusion, rigour and relevance. Full methodology including inclusion/exclusion criteria is reported elsewhere.^5 6 12^

This resulted in theories to explain how and why 13 types of behaviour change techniques or ‘strategies’ work to reduce or mitigate UB and what drives UB and how—reported separately elsewhere.^5 6^ Uniquely, this short report combines these two aspects of our analysis, whereby we mapped mechanisms underpinning drivers of UB^5^ against strategies which address these drivers^6^ to develop this overall explanatory PT.

## Results

**Figure 1:**
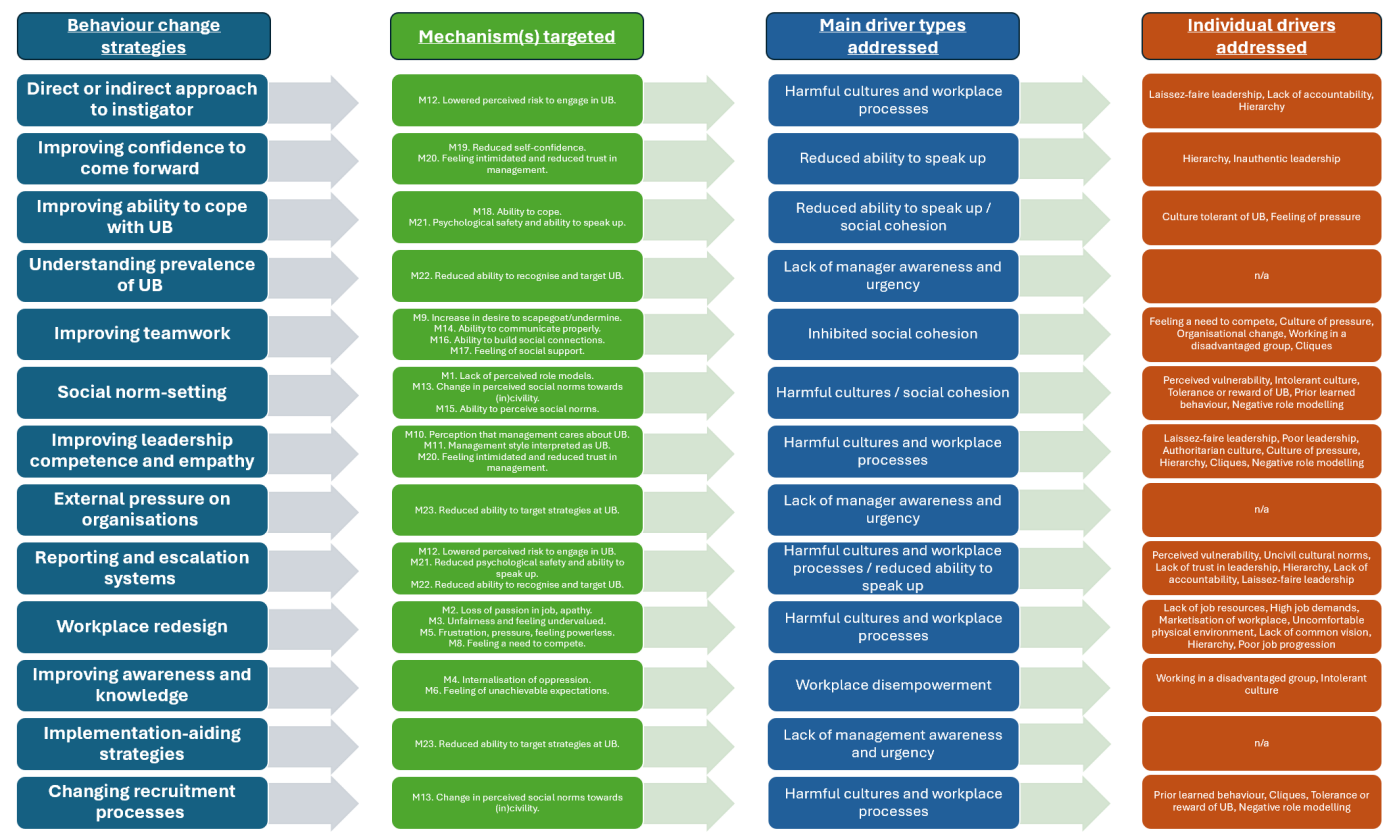
Diagram to depict which different behaviour change strategies target particular drivers of unprofessional behaviour (UB).

Our review encompassed 42 reports of interventions to address UB,^14–55^ 29 of which have been evaluated through various study designs. Figure 1 presents a PT diagram depicting which behaviour strategies target various mechanisms underlying drivers of UB, which driver categories are impacted by these strategies, and which individual drivers within these categories are targeted. This PT includes five major drivers of UB: (1) workplace disempowerment; (2) harmful workplace processes and cultures; (3) inhibited social cohesion; (4) a reduced ability to speak up and (5) lack of manager awareness and urgency.^5^ In table 1, we provide more details of these behaviour change strategies and how they target specific drivers of UB as well as how frequently each strategy type was used by the 29 included evaluated interventions. Online supplemental file 1 presents an alternative version of figure 1 designed specifically to map onto our PT published elsewhere and provides a further detailed version of table 1.^5^

10.1136/bmjoq-2024-002830.supp1 Supplementary data

**Table 1:** Matching the 13 types of strategy (and individual strategies within these) against types of drivers of UB

Primary driver addressed	Behaviour change strategies
Single incidents of UB (individual-level/does not address drivers)	Direct or indirect approach to instigator (target, bystander or managers)—used in 14 out of 29 evaluated interventions
	Informal resolution
	Disciplinary action
	Peer messengers
	Mediation
	Speaking up
Workplace disempowerment and staff ability to speak up	Improving confidence to come forward (target, bystander)—used in 22 out of 29 evaluated interventions
	Assertiveness training
	Role playing
	Cognitive rehearsal
	Keeping records
	Improving awareness and knowledge (all)—used in 12 out of 29 evaluated interventions
	Education, awareness and general group discussions
Improving social cohesion	Improving ability to cope with UB (target, bystander)—used in 0 out of 29 evaluated interventions
	Seeking help externally
	Journalling
	Moving targets
	Individual coping strategies
	Reflection
	Improving teamwork (all)—used in 16 out of 29 evaluated interventions
	Teambuilding exercises
	Conflict management training
	Communication training
	Journal club/group writing
	Problem-based learning
	Staff networks
Addressing harmful cultures and workplace processes	Social norm-setting (all)—used in 16 out of 29 evaluated interventions
	Championing
	Code of conduct
	Role modelling
	Environmental modification
	Allyship
	Improving leadership competence and empathy (managers/leaders)—used in 2 out of 29 evaluated interventions
	Leadership training
	Reverse mentoring
	Reporting and escalation systems (all)—used in 7 out of 29 evaluated interventions
	Reporting system
	Changing recruitment processes (all)—used in 0 out of 29 evaluated interventions
	Changing recruitment criteria
	Dismissal
	Workplace redesign (all)—used in 1 out of 29 evaluated interventions
	Democratisation of workplace
Improving manager awareness and urgency to address UB	External accreditation or pressure on organisations (managers/leaders)—used in 2 out of 29 evaluated interventions
	Seeking hospital Magnet status
	Regulator action
	Laws and regulations
	Understanding prevalence of UB (managers/leaders)—used in 3 out of 29 evaluated interventions
	Survey
	Multisource feedback
	Implementation-aiding strategies (managers/leaders)—used in 11 out of 29 evaluated interventions
	Action planning or goal setting
	Building a repertoire of strategies

UB, unprofessional behaviour.

Figure 1 highlights that many drivers of workplace disempowerment and harmful workplace processes are only addressed by workplace redesign strategies. Such workplace redesign strategies seek to facilitate staff autonomy, control and ownership of work; however, workplace redesign must occur at an organisational level and has only been used once in an evaluated intervention.^16^ Our work also shows that the most frequently used (often individual-focused) strategies, such as improving awareness and knowledge of UB, address few actual drivers of UB and therefore may not be as effective as other strategies.

## Discussion and conclusions

Existing interventions have made little use of logic models and behavioural science principles in their design, meaning that the rationale behind choice of behaviour change strategies has been poorly articulated and not evidence-based.^6^ Our PT, presented in figure 1, is a starting point to inform logic model design for those seeking to design evidence-based interventions that address particular drivers of UB.^56^ To improve reporting, future research should align and operationalise these strategies against existing Behaviour Change Technique (BCT) frameworks.^57^

Our PT has also highlighted that many systemic drivers remain under-addressed. Predominantly, existing interventions have focused on individual or team strategies to address UB with less focus on more systemic, potentially difficult-to-implement strategies such as redesigning the workplace to reduce frustrations and increase staff ownership over work.^6^

We have produced a free evidence-based guide for addressing UB in healthcare, available at https://workforceresearchsurrey.health/projects-resources/addressing-unprofessional-behaviours-between-healthcare-staff/.^58^
