# Supplementary material for: How can interventions more directly address drivers of unprofessional behaviour between healthcare staff?
Source: BMJ Open Qual. 2024 Jul 8;13(3):e002830. doi: 10.1136/bmjoq-2024-002830 (PMC11261740; doi:10.1136/bmjoq-2024-002830)
Supplement: Supplementary data [file bmjoq-2024-002830supp001.pdf]

Supplementary File 1. Alternative Figure 1 design and Table depicting which strategies address particular drivers of UB.

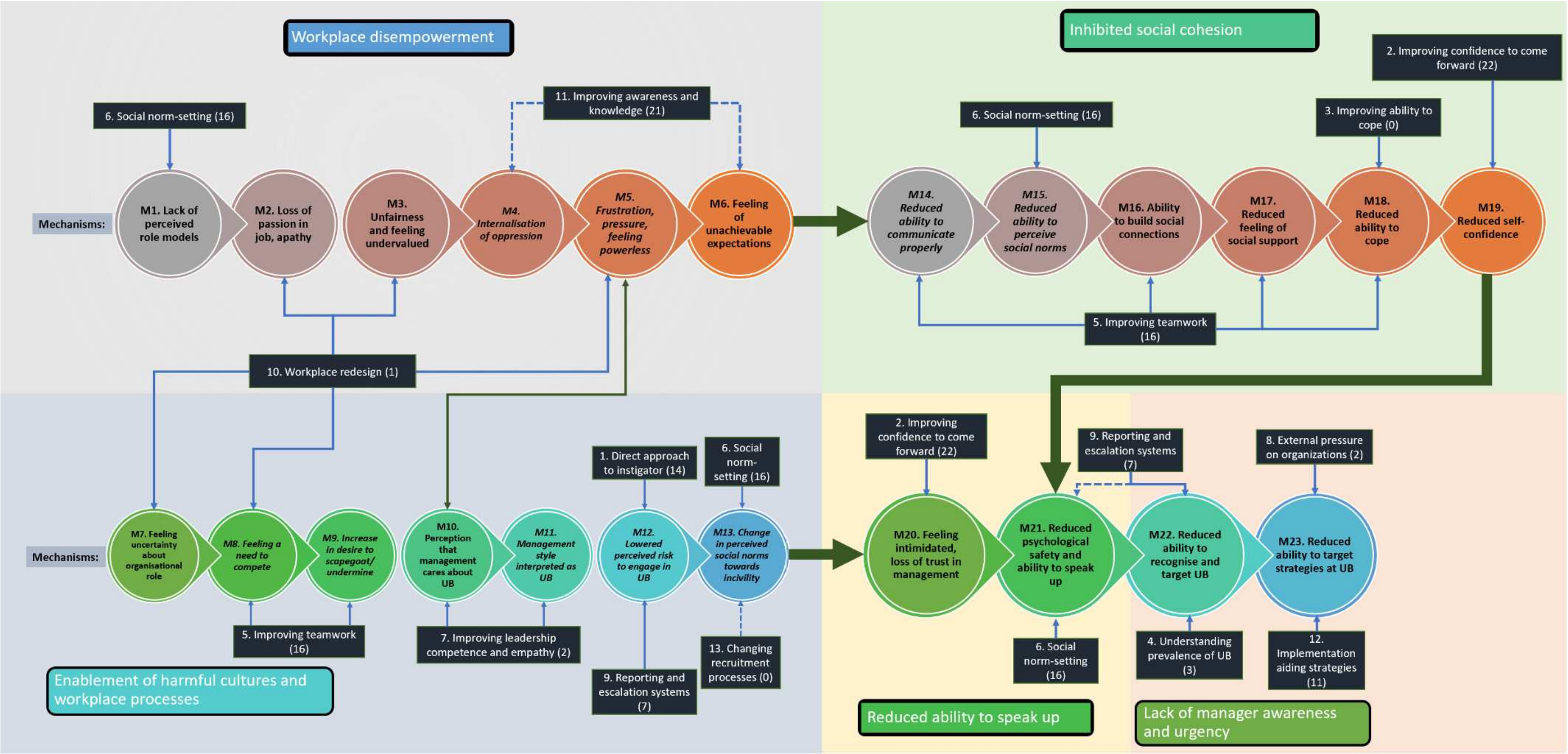

Figure 1. Diagram to depict which different behaviour change strategies target particular drivers of UB. Coloured areas indicate the category of driver these mechanisms affect (e.g. workplace disempowerment). Strategies are in black boxes and mechanisms targeted are in the circles. Dotted lines indicate connections with lesser evidence. The number in brackets after the strategy labels indicate the frequency with which a strategy has been

evaluated in one of the 42 included interventions in our review. Some strategies are depicted in multiple places because they target multiple drivers; for example, social norm-setting strategies (number 6) including positive role-modelling by leaders, can target the mechanism that drives UB, by influencing social norms away from negative behaviours. Likewise, social norm-setting strategies can enhance psychological safety by signalling a move towards a safer culture.

Table 1. Matching the thirteen types of strategy (and individual strategies within these) against types of drivers of UB.

Primary driver addressed	Behaviour change strategies	Description of strategy
**Single incidents of UB (individual-level/does not address drivers)**	**1. Direct or indirect approach to instigator (target, bystander, or managers) – used in 14 out of 29 evaluated interventions**	
	Informal resolution	Approach an instigator individually, or their line manager, to prompt reflection about behaviour, change future behaviour, or resolve situation.
	Disciplinary action	Staff who are reported to have behaved unprofessionally are called to a meeting with the human resources team or line manager. Disciplinary proceedings begin which may dissuade staff from repeating the behaviour.
	Peer messengers	Use of peer messengers is usually combined with a reporting system. Member of staff submits a report about an UB incident to a reporting system. Organisations send a specially trained peer messenger to have a conversation with the person who behaved inappropriately, to try to resolve the issue.
	Mediation	Brings the two parties (the person who behaved inappropriately and the person on the receiving end) together to resolve their differences. They are supported by a trained mediator who creates a safe environment for discussion. This is used in practice but not often used in interventions to change culture as it is individual focused and intensive.
	Speaking up	May involve the person stating in the moment that they are uncomfortable with the person's behaviour or it could involve reporting the UB to another such as a Freedom To

		Speak up Guardian or externally to a regulator or if all else fails to the media (whistleblowing). This approach requires staff to feel safe to speak up.
**Workplace disempowerment and staff ability to speak up**	**2. Improving confidence to come forward (target, bystander) – used in 22 out of 29 evaluated interventions**	
	Assertiveness training	Training helps boost staff members' self-confidence and assertiveness, to help them challenge unprofessional behaviours in real time.
	Role playing	Practising behaviours (such as speaking up) and self-reflection (such as those relating to poor self-esteem) in a group setting. It may enhance staff members' ability to cope or improve their confidence about coming forward.
	Cognitive rehearsal	Technique helps staff practise recognising unprofessional behaviours and using specific behaviours and thought patterns to help rehearse behaviours that improve coping or ability to come forward, and, if a situation occurs, to stop it from escalating.
	Keeping records	An individual strategy of recording or documenting incidences of unprofessional behaviours and details of the events, to better provide evidence if they raise a complaint.
	**11. Improving awareness and knowledge (all) – used in 12 out of 29 evaluated interventions**	
	Education, awareness and general group discussions	Delivering lectures or workshops to improve understanding of what UB are, how to recognise them, and how to informally address them in the moment. Usually used as a quick way to address unprofessional behaviours (although often insufficient on its own) or as a foundation for further intervention content.
	**3. Improving ability to cope with UB (target, bystander) – used in 0 out of 29 evaluated interventions**	

**Improving social cohesion**	Seeking help externally	Looking outside one's organisation for help – for example, contacting a union representative, regulatory body or GP. Individual strategy and an organisation can encourage this as needed, as part of a robust organisation-wide approach.
	Journalling	Reflective writing about one's experience of unprofessional behaviours in the workplace which may help with coping. Usually undertaken by individuals outside an intervention. However, organisations could encourage this as a coping strategy.
	Moving targets	Moving targets away from UB instigators in organisation. Should only be done with consent of the target and may just move problem elsewhere.
	Individual coping strategies	Includes various approaches that individuals may adopt themselves to help improve coping, such as breathing exercises, seeking therapy. Not suitable options for an organisational-level intervention or helpful on their own.
	Reflection	Engaging in self-reflection or group reflection activities such as in Schwartz Rounds [56].
	**5. Improving teamwork (all) – used in 16 out of 29 evaluated interventions**	
	Teambuilding exercises	Group sessions which incorporate activities to build a sense of social support and camaraderie.
	Conflict management training	Equips staff with the skills to de-escalate situations or prevent them from escalating them in the first place.
	Communication training	Enhances staff members' ability to communicate in a way that is less likely to be seen as unprofessional.
	Journal club / group writing	Writing in a group may help staff reflect on experiences of unprofessional behaviours and build social support.

	Problem-based learning	Group learning which involves identifying real-life problems and learning to tackle them. It often involves peer-to-peer teaching.
	Staff networks	Internal or external networks for staff from specific backgrounds (for example, members of ethnic minority communities, LBTQIA+ staff, or staff with disabilities) to share coping strategies and improve social support.
**Addressing harmful cultures and workplace processes**	**6. Social norm-setting (all) – used in 16 out of 29 evaluated interventions**	
	Championing	Gaining commitments from individuals to speak up about unprofessional behaviours and role model values and behaviours. Same individuals may also act as trusted contacts for reporting UB incidents.
	Code of conduct	Document that clarifies organisational policies on acceptable behaviour and processes to report or otherwise tackle UB.
	Role modelling	Similar to championing, leaders or managers adopt and demonstrate the behaviours and values they want to see / encourage in staff.
	Environmental modification	Modifying the physical environment to increase awareness of UB and expected conduct – for example, by putting up posters.
	Allyship	Staff who are less vulnerable to UB offer support to more marginalised colleagues and work to actively reduce inequalities.
	**7. Improving leadership competence and empathy (managers/leaders) – used in 2 out of 29 evaluated interventions**	

	Leadership training	Training improves staff members' management or communication styles and can help raise awareness and reduce bullying.
	Reverse mentoring	Enables staff in senior positions to learn from colleagues in more junior roles and come to understand issues from their perspective. It often involves staff from under-represented or marginalised groups. Not typically incorporated into culture change interventions as often small scale and time intensive.
	**9. Reporting and escalation systems (all) – used in 7 out of 29 evaluated interventions**	
	Reporting system	Reporting systems offer a means of reporting incidences of UB in the workplace. May be web-based or involve reporting to a specific person –named or anonymous. Can be anonymous or not. Examples include the Ethos system (Australia), and the Co-Worker Observation Reporting System from Vanderbilt University Medical Center (USA).
	**13. Changing recruitment processes (all) – used in 0 out of 29 evaluated interventions**	
	Changing recruitment criteria	Organisation changes its recruitment criteria to include personality or emotional intelligence tests or values-based recruitment. Can help organisations recruit staff who will flourish in a civil organisational culture.
	Dismissal	Dismissing an instigator known to have UB behaviour from employment.
	**10. Workplace redesign (all) – used in 1 out of 29 evaluated interventions**	
	Democratisation of workplace	Democratisation of workplace, e.g., staff representation on strategic committees, helping staff to feel heard.
	**8. External accreditation or pressure on organisations (managers/leaders) – used in 2 out of 29 evaluated interventions**	

**Improving manager awareness and urgency to address UB**	Seeking hospital Magnet status	Seeking 'Magnet status' or similar accreditations (more common in the USA), shows a hospital or organisation has a civil culture. Can lead to managers/ leaders becoming more focused on addressing a culture of incivility.
	Regulator action	Inspections by the CQC or other regulatory bodies may identify a culture of UB. This can place pressure on managers to tackle UB.
	Laws and regulations	Legislation may place responsibilities on organisations for ensuring equality and employee wellbeing and safety. This increases organisational urgency to address unprofessional behaviours.
	**4. Understanding prevalence of UB (managers/leaders) – used in 3 out of 29 evaluated interventions**	
	Survey	A survey can identify the level of UB in an organisation. May help target or design other strategies.
	Multisource feedback	Similar to reporting systems (see above). If someone has displayed UB, this approach investigates their behaviour from different staff members' perspectives, to provide a 360-degree view of behaviour over time.
	**12. Implementation-aiding strategies (managers/leaders) – used in 11 out of 29 evaluated interventions**	
	Action planning or goal setting	Action planning involves staff coming together to brainstorm and plan strategies to tackle UB. Using a co-creation approach helps staff feel heard and part of the solution to UB.
	Building a repertoire of strategies	Enables an organisation to be flexible in the interventions it delivers for tackling unprofessional behaviours. This improves organisational readiness for tackling different scenarios contributing to UB. This is used by the CREW intervention, for example.
